# Associations between a Genetic Risk Score for Clinical CAD and Early Stage Lesions in the Coronary Artery and the Aorta

**DOI:** 10.1371/journal.pone.0166994

**Published:** 2016-11-18

**Authors:** Elias L. Salfati, David M. Herrington, Themistocles L. Assimes

**Affiliations:** 1 Department of Medicine, Division of Cardiovascular Medicine, Stanford University School of Medicine, Stanford, California, United States of America; 2 Stanford Cardiovascular Institute, Stanford University, Stanford, California, United States of America; 3 Wake Forest University Baptist Medical Center, Medical Center Boulevard, Winston-Salem, North Carolina 27157, United States of America; Universite de Montreal, CANADA

## Abstract

**Objective:**

The correlation between the extent of fatty streaks, more advanced atherosclerotic lesions, and community rates of coronary artery disease (CAD) is substantially higher for the coronary artery compared to the aorta. We sought to determine whether a genetic basis contributes to these differences.

**Approach and Results:**

We conducted a cluster analysis of 6 subclinical atherosclerosis phenotypes documented in 564 white participants of the Pathobiological Determinants of Atherosclerosis in Youth study including the extent of fatty streaks and raised lesions in the coronary artery (CF and CR), thoracic aorta (TF and TR), and abdominal aorta (AF and AR) followed by a genetic association analysis of the same phenotypes. Our cluster analysis grouped all raised lesions and fatty streaks in the coronary into one cluster (CF, CR, TR, and AR) and the fatty streaks in the aorta into a second cluster (TF and AF). We found a genetic risk score of high-risk alleles at 57 susceptibility loci for CAD to be variably associated with the phenotypes in the first cluster (OR: 1.30 p = 0.009 for being in top quartile of degree of involvement of CF, 1.34 p = 0.005 for CR, 1.25: p = 0.11 for TR, and 1.19 p = 0.08 for AR) but not at all with the phenotypes in the second cluster (OR: 1.01, p = 0.95 for TF and 0.98, p = 0.82 for AF).

**Conclusions:**

The genetic determinants of fatty streaks in the aorta do not appear to overlap substantially with the genetic determinants of fatty streaks in the coronary as well as raised lesions in both the coronary and the aorta. These findings may explain why a larger fraction of fatty streaks in the aorta are less likely to progress to raised lesions compared to the coronary artery.

## Introduction

Autopsy studies suggest that the fatty streak is the earliest identifiable lesion of atherosclerosis. However, not all fatty streaks progress to more advanced lesions of atherosclerosis[1]. Furthermore, the correlation between the extent of fatty streaks in younger persons and the extent of more advanced lesions in older persons as well as the rates of CAD in communities within which these autopsy studies were performed is substantially lower for the aorta compared to the coronaries[2, 3]. These observations suggest that the pathophysiologic processes leading to the formation of fatty streaks may differ between the coronary and aortic vascular beds.

We have previously shown that a genetic risk score (GRS) summarizing an individual's exposure to high risk alleles at 49 susceptibility loci for clinical CAD predicts the presence and extent of early and uncomplicated raised lesions in the right coronary artery (RCA) of adolescent and young adults aged 15 to 34 at the time of autopsy in the NHLBI sponsored Pathobiological Determinants of Atherosclerosis in Youth (PDAY) study[4]. If fatty streaks are uniformly necessary precursors to early stage raised atherosclerotic lesions, we would expect that a GRS of CAD would not only be associated with the degree of early raised lesions in the coronary but also with the degree of fatty streaks in the coronary. If the CAD loci included in this GRS additionally largely represent loci that promote atherosclerosis more generally across all arterial beds, then the GRS would be expected to also associate with fatty streaks and raised lesions in other arterial beds.

The PDAY study provides an opportunity to compare and contrast the magnitude of association between a GRS and early stage lesions in the RCA to that of a GRS and early stage lesions in the aorta. Such comparisons have not been previously reported and could shed light on the similarities and differences in the pathogenetic processes responsible for the formation of fatty streaks and raised lesions in different vascular beds. In this context, the main objective of this study was to identify similarities and differences of six phenotypes documented in PDAY including the degree of fatty streaks and raised lesions in the RCA, thoracic aorta, and abdominal aorta through a cluster analysis, and to determine whether the result of the cluster analysis is consistent with the degree of association observed between a GRS of CAD and the same six phenotypes. Secondary objectives included i. documenting whether GRS associations we observe persists even after the exclusion of risk-factor SNPs and ii. testing an improved GRS that includes 8 recently reported novel susceptibility loci of CAD as well as improved imputation through the use of the Haplotype Reference consortium resource [5, 6].

## Material and Methods

### Study Population

The Pathobiological Determinants of Atherosclerosis in Youth (PDAY) is a unique NHLBI repository that includes of a total of 2876 study subjects, between 15 and 34 years old, black and white, men and women, who died of external, non-atherosclerosis related causes and underwent autopsy between June 1, 1987, and August 31, 1994. All subjects had post-mortem quantitative assessment and documentation of the degree of early atherosclerosis including the percent (%) surface area of fatty streaks and raised lesions in the right coronary artery, thoracic aorta and abdominal aorta. Detailed descriptions of the design and methods for the PDAY study have been published elsewhere[3].

### Phenotyping

Pathologists, blinded to demographic, clinical, and pathological observations, evaluated the right coronary arteries and left halves of the aortas. They visually estimated the extent of intimal surface involved with fatty streaks, fibrous plaques, complicated lesions, and calcified lesions by procedures developed in the International Atherosclerosis Project[3]. A fatty streak was a flat or slightly elevated intimal lesion stained by Sudan IV and without other underlying changes. A fibrous plaque was a firm, elevated, intimal lesion, sometimes partially or completely covered by sudanophilic deposits. A complicated lesion was a plaque with hemorrhage, thrombosis, or ulceration. A calcified lesion was an area in which calcium was detectable, either visually or by palpation, without overlying hemorrhage, ulceration, or thrombus. The sum of the percentages of surface involved with fibrous plaques, complicated lesions, and calcified lesions by gross visual grading was designated "raised lesions." Raised lesions were predominantly fibrous plaques. Consensus grading of lesions was the average of independent gradings by three pathologists. Intraobserver variability was assessed by repeated independent gradings of coded specimens randomly interspersed among new specimens[3].

### Genotyping and Imputation

The SNPs and the Extent of Atherosclerosis (SEA) study was launched in 2009 to identify genetic variants associated with premature atherosclerosis in subjects included in PDAY. One of the main goals of SEA study was to use the quantitative measures of atherosclerotic lesions in the PDAY cohort as the target phenotype for a genome-wide association study. Because of financial constraints, not all 2876 samples could be genotyped. In an attempt to maximize power to discover variants, SEA adopted a pseudo “extreme phenotype” approach to sampling with the phenotype of interest being the mean degree of subclinical raised lesions in both coronary and aortic vascular beds observed in PDAY subjects [4]. Among all subjects with at least some raised lesions in either the coronary or aortic beds, 357 subjects with the largest positive residual from the mean level of raised lesions predicted by a regression model adjusting for age, age^2^, sex, and race were selected for genotyping. Thereafter, an additional 711 subjects among all subjects with a zero (or near zero) mean raised lesion score were also selected for genotyping. These 711 subjects were matched on age, gender, and race to the 357 subjects with raised lesions. Both European and African American subjects were included in the SEA study but analyses in this study are restricted to the subset of 564 subjects of European ancestry. This restriction was imposed because the CAD susceptibility SNPs we used in the construction of our GRS all have been uncovered in European populations.

Genotyping for the SEA study began in ~2009 and progressed in two phases. In Phase I, quantitative genotyping was used to estimate allele frequency for >2.4 million SNPs in each of eight pools of DNA (3 case and 5 control pools) using one of the first arrays designed by Perlegen Sciences with genome wide coverage. Pools were balanced for age, gender and genetic ancestry based on analysis of 311 ancestry informative markers typed on every sample before pool formation. In Phase 2, the 357 subjects with raised lesions and 711 subjects matched controls described above were genotyped at the individual level with a Perlegen array that included approximately 106,285 SNPs across the genome.

We obtained both the genetic data generated by SEA investigators and the phenotypic data generated by PDAY to conduct this study from the NCBI's database of Genotypes and Phenotypes (dbGaP)[7]. The Institutional Review Board at Stanford University approved the use of data from dbGaP to conduct this study. Data Supplement (Table A in S1 File) lists accession numbers and embargo dates for all dbGaP files used.

The small size of the array necessitated imputation of SNPs to minimize the need to search for proxies when constructing our GRSs. Previously, we imputed the SNPs in the 1000 Genomes Project reference haplotypes using MaCH (v1.0.18.c) and Minimac (2013-07-17) software following a standard two-stage imputation procedure[4]. Here, we performed a second round of imputation using a reference panel of 64,976 haplotypes at 39,235,157 SNPs constructed using whole genome sequence data from 20 studies of predominantly European ancestry, which is ~33 times larger than the size of the 1000 Genome Project reference panel[6]. We carried out this imputation and phasing using remote server resources from the University of Michigan at https://imputationserver.sph.umich.edu/start.html

### Selection of SNPs and Construction of the GRS

Our baseline GRS was constructed from 49 SNPs (LD-pruned, r2 < 0.2) included in Supplementary Table 9 of the 2013 CARDIOGRAMplusC4D report that had reached genome-wide significance for clinical CAD at any time during the GWAS era[8]. To this GRS, we added the lead SNPs at 8 new loci identified by 1000 Genomes-based genome-wide association meta-analysis of CAD[5]. As expected, none of the 57 SNPs were genotyped in the original Perlegen array. We calculated a weighted multi-locus GRS for each individual using their imputed genotype dosage of the number of high-risk alleles for 57 SNPs (wGRS-57). An extensive description of the calculation of a weighted multi-locus GRS can be found elsewhere[4, 9].

The CARDIoGRAM+C4D consortium reported that 13 of the 57 SNPs for CAD showed either genome wide significance or strong trends for association for lipid phenotypes and 6 for blood pressure phenotypes. To assess whether any of our associations were being driven by traditional risk factors, we created and tested a second weighted GRS restricted to the remaining 38 SNPs (wGRS-38) demonstrating no clear association with traditional risk factors.

We applied three imputation quality cutoffs to each of these two wGRSs to create 6 more wGRS restricted to SNPs with an imputation r^2^ > 0.3, 0.5, and 0.8 respectively. Lastly, we created analogous wGRSs with the imputations from the HRC in lieu of the imputations derived from the 1000G-reference panel.

### Case Definition

We stratified individuals by sex and then ranked them in decreasing order by the percent surface area (% SA) of "raised lesions" present in the right coronary artery (CR), the thoracic aorta (TR), and abdominal aorta (AR). Similarly, we ranked all individuals by the % SA of “fatty streaks” present in the same three locations (CF, TF, AF). Thus, each individual was assigned a sex-specific % SA ranking for 6 phenotypes. For each phenotype, subjects within the top 25^th^ percentile were defined as cases and the remaining subjects were defined as controls. Subjects could serve as a case for some phenotypes and a control for others if their sex-specific % SA ranking was above the 25^th^ percentile for some phenotypes and below for other phenotypes. For the thoracic aorta, we had to slightly modify the case definition for raised lesions (TR) because less than 25% of subjects had any raised lesions in this location. Thus, cases were defined as individuals with any raised lesions for this phenotype and the remaining subjects with no raised lesions in this region were assigned as controls.

### Statistical Analysis

We first examined the global correlation patterns of the six phenotypes of interest (percentage of surface area of involvement with “raised lesions” and “fatty streaks” in the coronary artery, thoracic aorta, and abdominal aorta, respectively) using two different clustering algorithms: Ward’s hierarchical clustering procedure and a k-means clustering procedure implemented in the R package “cluster”[10, 11]. To assess the effect of the oversampling of PDAY subjects with advanced raised lesions in all three vascular beds, we repeated the Ward’s hierarchical clustering procedure on all white PDAY participants.

Next, we used logistic regression to estimate the association between a GRS and case-control status of the six phenotypes as defined above. In addition to the use of sex-specific score cutoffs to define cases, we further adjusted our models for age and sex to account for any potential residual confounding related to these variables. We calculated the power to detect an OR of 1.3 for our sample size and proportion of cases using the method by Hsieh et al. incorporated in PASS 11.0 (NCSS, Kaysville, UT, USA). Lastly, we assessed whether the difference between each of the three pairs of beta coefficients within a lesion type was significantly different than zero taking into consideration correlations between the phenotypes[12]. If no differences were found between the three pairs within a lesion type, we calculated the mean effect for the GRS for that lesion once again taking into consideration the correlation of phenotypes between the three pairs[12].

We performed three sets of sensitivity and/or subgroup analyses. The first involved recomputing GRS associations after successively removing SNPs with low imputations scores (imputation cutoffs considered were r^2^> 0.3, r^2^>0.5, and r^2^>0.8). The second involved computing GRS associations in 2 subgroup defined by the median age the time of autopsy. The final involved performing SNP by SNP association analyses followed by a meta-analysis of all 57 SNPs for each phenotype.

## Results

We examined a total of 564 PDAY participants of white/European descent who underwent individual level genotyping through Phase II of the SEA study. The mean (SD) of these subjects at the time of death was 26.7 (5.0) and 22.7% of them were female. We used a total of 66,166 SNPs on the Perlegen array that passed quality control to perform our imputation with the 1000G-reference panel. The average r^2^ for the 57 SNPs used to construct our genetic risk scores was 0.26 with 35.1% of these SNPs having an imputation r^2^ > 0.3. The mean and standard deviations of our wGRS-57 and wGRS-38 were 3.5 (0.2) and 2.7 (0.2), respectively.

Table 1 provides summary statistics for the six phenotypes of interest in PDAY. The proportion of subjects with no fatty streaks was notably higher in the coronary vascular bed compared to the aortic vascular. However, the same trend was not obvious for raised lesions. The proportion of subjects defined as cases was close to the expected 25% for five out of our six phenotypes. The sole exception was for raised lesions in the thoracic aorta where only 10.3% were found to have any raised lesions.

**Table 1:** Summary statistics for the six phenotypes of interest in 564 white/European genotyped participants of the Pathobiological Determinants of Youth study.

Lesion Type	Vascular Bed	No. (%) with SA* = 0	Cut off to identify top quartile of % SA* of involvement		No. (%) of Cases	Mean % SA*	
			Females	Males		Cases	Controls
Fatty Streaks	Abdominal Aorta	0 (0)	52.3	34.2	141 (25)	52.6	18.7
	Thoracic Aorta	1 (0.2)	25.2	25.7	137 (24.3)	37.1	13.3
	Right Coronary Artery	203 (36)	3.7	4.4	140 (24.8)	13.4	1.0
Raised Lesions	Abdominal Aorta	362 (64.2)	8.9	2.3	141 (25)	19.5	0.23
	Thoracic Aorta	506 (89.7)	0.0	0.0	58 (10.3)	3.7	0.0
	Right Coronary Artery	383 (67.9)	0.43	1.6	140 (24.8)	16.3	0.07

*SA: Surface Area

**Fig 1:**
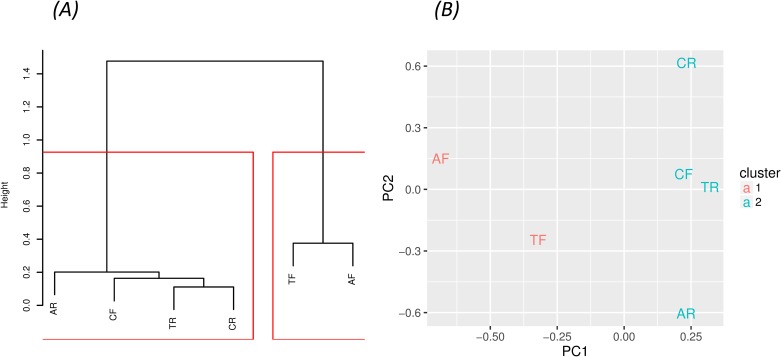
Cluster analysis of six phenotypes in 564 white participants of the Pathobiological Determinants of the Youth (PDAY) study including percent surface area of involvement by raised lesions and fatty streaks in the coronary (CR and TF), thoracic aorta (TR and TF), and abdominal aorta (AR and TF). Both clustering algorithms identified 2 clusters. The first cluster includes CF, CR, TR, and AR and the second cluster includes TF and AF.

Our two cluster analyses of the six phenotypes of interest is summarized in Fig 1. Both algorithms grouped all raised lesions and fatty streaks in the coronary into one cluster (CF, CR, TR, and AR) and the fatty streaks in the aorta into a second cluster (TF and AF) into a second cluster. When requesting a third cluster, both algorithms split the second cluster into 1 cluster for TF alone and a second cluster for AF alone (details not shown). We found the clustering to be identical when we repeated the analyses in all 1344 white PDAY participants (Figure A in S1 File). Thus, the clustering was not driven by the oversampling of subjects with advanced raised lesions adopted by SEA.

**Fig 2:**
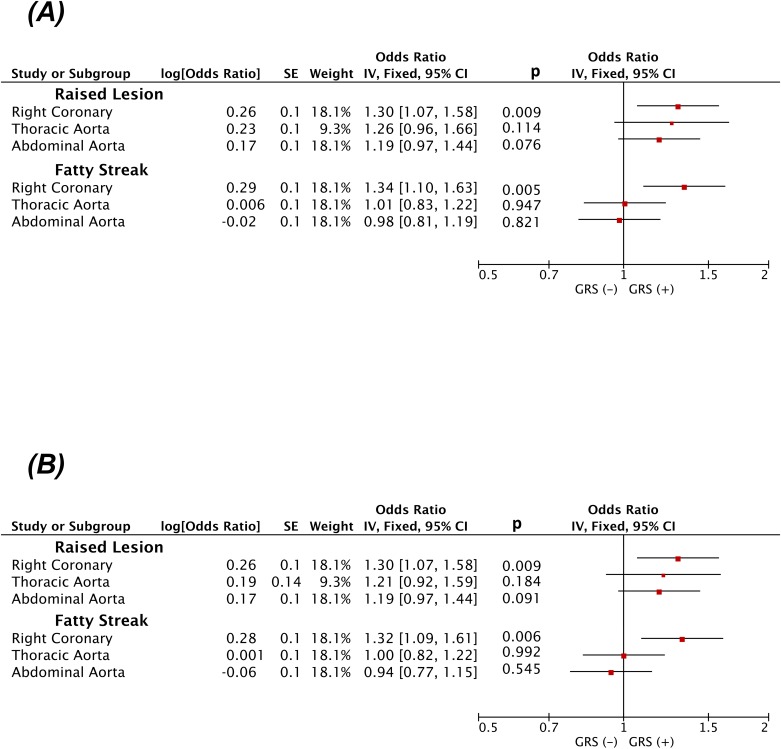
Forest plot of Odds Ratio per standard deviation increases in the weighted GRS (wGRS) for being in the top quartile of percent surface area of involvement (case) of early arterial lesions compared to the bottom three quartiles (controls). Odds Ratios are adjusted for age and sex at time of autopsy. Results for all 3 vascular beds are grouped by lesion type (raised lesions vs. fatty streak) and shown for wGRS that includes all 57 SNPs associated with clinical CAD (A) and for the subset of 38 SNPs not associated with traditional risk factors (B).

Fig 2 summarizes our association results between each of our two weighted GRSs and case-control status. We found a wGRS of 57 high-risk alleles for clinical CAD to be associated with ~30% increased risk of being in the top quartile of the degree of raised lesions in the RCA (p = 0.009). The power to detect this degree of increased risk was 77% assuming an α = 0.05. The magnitude of association and the statistical significance of this finding were comparable to those observed for fatty streaks in the RCA. The next two most significant associations observed were for raised lesions in the abdominal and the thoracic aorta with p values that showed a trend towards significance (p = 0.08 and 0.11, respectively). Lastly, we observed no association between the wGRS57 and fatty streaks in both the thoracic and abdominal aorta (p >0.82). The OR for coronary fatty streaks was significantly different than the OR for thoracic and abdominal aortic fatty streaks (p = 0.02 and 0.01, respectively) but there was no differences in the ORs between any of the associations calculated for raised lesions. The mean OR of the GRS for raised lesions across all three sites was 1.25 (p = 0.003).

All of these findings and trends were similar for our wGRS38 restricted to SNPs not associated with traditional risk factors of CAD (Fig 2). We could not detect any statistically significant association between a GRS restricted to 19 risk-factor SNPs and any of the six phenotypes (Table B in S1 File). However, the same was true when we tested a randomly selected 19 SNPs among either all 57 SNPs or among the 38 non risk factor SNPs (Tables C and D in S1 File).

Tables E and F in S1 File summarize the association results for each of six phenotypes when using wGRS restricted to SNPs with imputation r^2^> 0.3, r^2^>0.5, and r^2^>0.8. Restricting the GRS to the subset of SNPs with a higher imputation score resulted in an appreciably stronger signal only for lesions in the RCA and only at an imputation r^2^ threshold of 0.3 that restricted the GRS to 20 SNPs.

Figure B in S1 File illustrates that the estimated imputation r^2^ of most of the 57 SNPs was modestly higher when SNPs were imputed using the HRC reference panel compared to the 1000G-reference panel (mean r^2^ = 0.42 vs. r^2^ = 0.26) although 2 SNPs could not be imputed at all. However, this improvement in imputation had no appreciable effects on any of our association analyses between our wGRS and case-control status (Table G in S1 File).

We did not detect an obvious stronger association between the GRS and fatty streaks in the younger age group (15 to 27 years) for the aorta (Figure C in S1 File). The point estimates for fatty streaks in the coronary were comparable between the two age groups. The signal for raised lesions appeared stronger across all beds among the older age group (Figure C in S1 File).

A meta-analysis of individual SNP estimates of effect yielded levels of statistical significance that were comparable to our results using a GRS (Tables H-M in S1 File). A clear single dominant SNP in terms of statistical association with all 6 phenotypes did not emerge although SNPs in the 9p21 region were the most significant for CF, in the 6^th^ position for CR, and in the 3^rd^ position for AR (Tables I, H and L in S1 File).

## Discussion

We found the formation of fatty streaks in the RCA of PDAY participants to be more closely related to the formation of raised lesions in both the coronary and the aorta than to the formation of fatty streaks in aorta. This relationship was evident not only in our cluster analyses of the six phenotypes but also in our genetic association analysis with each of the six phenotypes. Indeed, the magnitude of association between a wGRS of clinical CAD susceptibility loci and fatty streaks was remarkably heterogeneous with a signal in the RCA that was comparable in magnitude to the strongest signal observed overall for raised lesions in the same vascular bed. In contrast, the signal for fatty streaks in either of the two locations of the aorta was completely absent. Our findings support the notion that not all fatty streaks identified through the gross examination of arterial vessels are equivalent in their predisposition to atherosclerosis. Fatty streaks in the aorta as a whole appear to be genetically less inclined to progress to raised lesions compared to the coronary.

We found no discernable effect on the strength of all our genetic associations when we restricted our GRS to 38 SNPs least likely to be operating through traditional risk factors. When we tested the 19 risk factors SNPs alone in a GRS, we found no significant associations for any of the 6 phenotypes but the levels of significance mirrored those achieved for a random subset of 19 SNPs, and thus could be purely a consequence of lack of statistical power. Consequently, we can only conclude that the risk-factor SNPs are not primarily responsible for the associations observed given the results remain significant with the GRS38. The risk-factor SNPs may still be contributing to the association when they are included in the GRS57.

We observed the largest source of heterogeneity in effect sizes among our tests of association for fatty streaks. While less heterogeneity was observed among our tests of association for raised lesions at the three vascular sites, the strongest association was undoubtedly observed for the RCA. The RCA was also the only phenotype whose strength of association improved when restricting the GRS to SNPs with an r^2^ > 0.3. These observations are consistent with the possibility that some of the CAD susceptibility loci included in our GRS may be influencing the development of plaque exclusively in the coronary tree. Interestingly, at least one of the CAD susceptibility loci includes a transcription factor, *TCF21*, which has been shown to have a specific role in the origin of the coronary artery smooth muscle cells and cardiac fibroblasts[13]. While *TCF21* is expressed in the vasculature of other developing organs, including the kidney and lung, it is not expressed in the aorta[14]. Furthermore, *TCF21* is one of the few CAD loci operating completely independent of traditional risk factors that has not yet been associated with atherosclerosis in non-coronary vascular beds[15].

We found that the addition of 8 new loci to our GRS had no discernable effect on the strength of association between the GRS and raised lesions in the RCA. Similarly, the addition of the new loci had no discernable effect on any of the other five phenotypes we examine for the first time in this study (data not shown). Whether this observation reflects differences in the mechanism of predisposition to clinical CAD between the eight new loci and the 49 previously discovered loci remains unclear. More likely, these findings reflect low power to detect an association due to a combination of a small number of new SNPs, small effect sizes, and overall suboptimal imputation.

We have previously hypothesized that the strength of our association between a GRS of clinical CAD and raised lesions in the RCA may be substantially underestimated because of the low quality of our imputation which would be expected to be non-differential between cases and controls[4]. We tested this hypothesis by re-imputing all SNPs using a reference panel approximately 33 times the size of the 1000 genomes panel. While we observed an improvement in the imputation quality for almost all SNPs, the improvements were modest and there were no discernible effects of these improvements on the magnitude and strength of the genetic associations we observed. The most effective way to test this hypothesis remains to either directly genotype or sequence the SNPs of interest or impute them after genotyping samples with contemporary genotyping chips that offer much higher genomic coverage for common variants compared to the first generation Perlegen array.

These observations are consistent with the previously well-documented differences in the prevalence, the time of appearance, and progression of fatty streaks in the aorta compared to coronary arteries[16]. For example, nearly all children over the age of 3 have some degree of aortic fatty streaks and the extent of aortic fatty streaks in the aorta differs minimally between children and adolescents of all populations irrespective of the degree of more advanced atherosclerotic lesions in adults from the same populations[17, 18]. On the other hand, fatty streaks appear in the coronaries about 5 to 10 years after they appear in the aorta and are located much more commonly in the regions where raised lesions are observed in older persons[18–20].

The universal presence of fatty streaks in the aorta of young children has fostered skepticism on its role in the formation of atherosclerosis for many years after it was first observed in the 1950s[16]. However, more recent extensive chemical, physiochemical, histologic, and electron microscopic studies as well as macroscopic examination of fatty streaks has led to the identification and characterization of two subtypes of fatty streaks: the “flat fatty streak” which is not elevated above the intimal surface of the artery and contains foams cells with minimal extracellular lipid deposits or connective tissue, and the “raised fatty streak” which is elevated above the surrounding surface and possesses not only foam cells but also pools of lipids in the extracellular space that is otherwise devoid of live cells[21, 22]. The raised fatty streak most likely represents a distinct lesion transitioning to a raised plaque. Indeed, the raised fatty streak was described and incorporated into the AHA’s report from the Committee on vascular lesions as a distinct intermediate “Type III” lesion possessing a higher probability of progression to atheroma than a Type II lesion (fatty streak)[1, 23]. In PDAY, the proportion of Type III lesions among all fatty streaks has been previously reported to be in the range of 30–35% in the RCA compared to only 5–10% in the aorta[24]. This difference in proportion may at least in part explain why our GRS associates with fatty streaks in the coronary and not the aorta. Unfortunately, we could not test this hypothesis directly because the “raised fatty streak” phenotype in PDAY is not available in dbGaP. A larger fraction of the flat fatty streaks in the coronary artery may also be destined to become Type III, and, eventually, Type IV “raised lesions” when compared to the aorta. Through a stratified subgroup analysis by the median age, we explored whether the atherosclerosis prone fatty streaks in the aorta could be better detected among younger subjects with overall less fatty streaks at autopsy but could not detect any obvious trends to support this hypothesis.

The probability of a fatty streak transitioning to a Type III and Type IV raised lesion may also be rooted in distinct differences of the embryological origin, function and anatomy of the coronaries compared to the aorta. Recent studies of embryos and fetuses confirm that the development of the coronary arteries in humans is similar to other mammalian and avian species[25, 26]. Specifically, the ostia and stems of the coronary arteries appear to be formed by the growth of a vascular plexus into the aorta that originates from the mesenchymal-containing atrioventricular cushions over the interventricular groove. Thus, the aortic system develops first and the coronary arteries follow with the coronary stems penetrating the root of the aorta rather than the entire system developing from an outgrowth of the aorta. Other gross histologic and anatomic differences between the two vascular beds that could be influencing the propensity for fatty streaks to progress to raised lesions include the more muscular and less elastic wall, the greater volume of thickened intima, and the larger number of branches of the coronaries compared to the aorta[27, 28].

In summary, a GRS of 57 high-risk alleles at established susceptibility loci for CAD predicts the presence of raised lesions in both the coronary and aortic beds but only fatty streaks in the coronary bed. The degree of associations are consistent with a cluster analysis of these phenotypes which clustered all raised lesions and coronary fatty streaks in one cluster and aortic fatty streaks in a second cluster. The findings suggest the presence of pathogenetic differences between fatty streaks in the coronary and those in the aorta that may contribute to differences in the predisposition of fatty streaks to progress to raised atherosclerosis lesions. The less robust associations of the GRS with raised lesions in the aorta compared to the coronary points to the possibility that at least some of the susceptibility loci for CAD operate exclusively in the coronary tree. However, more research is needed to draw more definitive conclusions including repeated association analyses after a more complete and reliable assessment of genetic variation in the PDAY samples using contemporary genotyping and/or sequencing technologies.

## Supporting Information

S1 File **Table A in S1 File.** List of variables used in this study, file names, and embargo dates from the database of genotypes and phenotypes (dbGAP). **Table B in S1 File.** Association between case-control status of raised lesions or fatty streak for each vascular beds and a weighted GRS composed of 19 SNPs associated with traditional risk factors adjusting for age and sex. **Table C in S1 File.** Association between case-control status of raised lesions or fatty streak for each vascular beds and a weighted GRS composed of 19 random SNPs from the 57 SNPs associated with clinical CAD and traditional risk factors adjusting for age and sex. **Table D in S1 File.** Association between case-control status of raised lesions or fatty streak for each vascular beds and a weighted GRS composed of 19 random SNPs from the 38 SNPs associated with clinical CAD only adjusting for age and sex. **Table E in S1 File.** Association a weighted GRS of 57 SNPs associated with clinical coronary artery disease and case-control status after further filtering by imputation quality r^2^ (0.3; 0.5; 0.8). **Table F in S1 File.** Association a weighted GRS restricted to 38 SNPs not associated with traditional risk factors and case-control status after further filtering by imputation quality r^2^ (0.3; 0.5; 0.8). **Table G in S1 File.** Association between weighted GRS and case-control status when using genotypes imputed with the Haplotype Reference Consortium. **Table H in S1 File.** Age and sex adjusted association with case-control status of right coronary raised lesions for each of the 57 single nucleotide polymorphisms used to generate the weighted genetic risk score, ranked by p-value from lowest to highest. **Table I in S1 File.** Age and sex adjusted association with case-control status of right coronary fatty streak for each of the 57 single nucleotide polymorphisms used to generate the weighted genetic risk score, ranked by p-value from lowest to highest. **Table J in S1 File.** Age and sex adjusted association with case-control status of thoracic aorta raised lesions for each of the 57 single nucleotide polymorphisms used to generate the weighted genetic risk score, ranked by p-value from lowest to highest. **Table K in S1 File.** Age and sex adjusted association with case-control status of thoracic aorta fatty streak for each of the 57 single nucleotide polymorphisms used to generate the weighted genetic risk score, ranked by p-value from lowest to highest. **Table L in S1 File.** Age and sex adjusted association with case-control status of abdominal aorta raised lesions for each of the 57 single nucleotide polymorphisms used to generate the weighted genetic risk score, ranked by p-value from lowest to highest. **Table M in S1 File.** Age and sex adjusted association with case-control status of abdominal aorta fatty streak for each of the 57 single nucleotide polymorphisms used to generate the weighted genetic risk score, ranked by p-value from lowest to highest. **Figure A in S1 File.** Ward’s hierarchical clustering analysis (using JMP Genomics SAS) of six phenotypes in all white participants of the Pathobiological Determinants of the Youth study (n = 1344) including percent surface area of involvement by raised lesions and fatty streaks in the coronary (CR and TF), thoracic aorta (TR and TF), and abdominal aorta (AR and TF). The algorithms identified 2 clusters. The first cluster includes CF, CR, TR, and AR and the second cluster includes TF and AF. The clusters identified are the same as those identified in the subset of 564 subjects selected for genotyping by SEA investigators. **Figure B in S1 File. Comparison of the quality of imputation between 1000 genomes reference panel (red) and the Haplotype Reference Consortium panel (blue) for each of the 57 SNPs used to construct our weighted genetic risk scores.** The y-axis plots the estimated imputation accuracy (r2). The x-axis lists the SNPs in order of increasing imputation r2 when using the 1000 genomes reference panel. Two SNPs could not be imputed using the Haplotype Reference Consortium panel. **Figure C in S1 File.** Forest plot of Odds Ratio per standard deviation increases in the weighted GRS (wGRS) for being in the top quartile of percent surface area of involvement (case) of early arterial lesions compared to the bottom three quartiles (controls). Odds Ratios are adjusted for age and sex at time of autopsy. Results for all 3 vascular beds are grouped by lesion type (fatty streak vs. raised lesions) and by age group (young 15-27yo vs. old 28-35yo) and shown for wGRS that includes all 57 SNPs associated with clinical CAD.
(PDF)
