# Supplementary material for: Associations between a Genetic Risk Score for Clinical CAD and Early Stage Lesions in the Coronary Artery and the Aorta
Source: PLoS One. 2016 Nov 18;11(11):e0166994. doi: 10.1371/journal.pone.0166994 (PMC5115850; doi:10.1371/journal.pone.0166994)

Table A. List of variables used in this study, file names, and embargo dates from the database of genotypes and phenotypes (dbGAP).

Dataset Accession	Variable accession	Variable name	Variable description	Embargo Release Date
pht002191.v1.p1	phv00159041.v1.p1	seaid	SEA STUDY RECODED ID	5/5/11
pht002191.v1.p1	phv00159042.v1.p1	sex	SEX	5/5/11
pht002191.v1.p1	phv00159043.v1.p1	race	RACE	5/5/11
pht002191.v1.p1	phv00159044.v1.p1	age	AGE	5/5/11
pht002191.v1.p1	phv00159047.v1.p1	tf	THORACIC AORTA FATTY STREAKS (% SURFACE AREA)	5/5/11
pht002191.v1.p1	phv00159048.v1.p1	tr	THORACIC AORTA RAISED LESIONS (% SURFACE AREA)	5/5/11
pht002191.v1.p1	phv00159049.v1.p1	af	ABDOMINAL AORTA FATTY STREAKS (% SURFACE AREA)	5/5/11
pht002191.v1.p1	phv00159050.v1.p1	ar	ABDOMINAL AORTA RAISED LESIONS (% SURFACE AREA)	5/5/11
pht002191.v1.p1	phv00159051.v1.p1	cf	RIGHT CORONARY ARTERY FATTY STREAKS (% SURFACE AREA)	5/5/11
pht002191.v1.p1	phv00159052.v1.p1	cr	RIGHT CORONARY ARTERY RAISED LESIONS (% SURFACE AREA)	5/5/11

Table B. Association between case-control status of raised lesions or fatty streak for each vascular beds and a weighted GRS composed of 19 SNPs associated with traditional risk factors adjusting for age and sex.

wGRS (=19 SNPs)	Estimate	SE	Z-value	P	Lesions
Right Coronary Artery	0.05376	0.09996	0.538	0.591	
Thoracic Aorta	0.13738	0.14219	0.966	0.334	**Raised Lesions**
Abdominal Aorta	0.05552	0.09991	0.556	0.578	
Right Coronary Artery	0.07866	0.1014	0.776	0.438	
Thoracic Aorta	0.01481	0.09953	0.149	0.8817	**Fatty Streak**
Abdominal Aorta	0.09561	0.09965	0.959	0.337312	

Table C. Association between case-control status of raised lesions or fatty streak for each vascular beds and a weighted GRS composed of 19 random SNPs from the 57 SNPs associated with clinical CAD and traditional risk factors adjusting for age and sex.

wGRS (=random 19 SNPs form 57 SNPs)	Estimate	SE	Z-value	P	Lesions
Right Coronary Artery	0.16353	0.09834	1.663	0.0963	
Thoracic Aorta	-0.09597	0.13875	-0.692	0.4891	**Raised Lesions**
Abdominal Aorta	-0.05037	0.09838	-0.512	0.609	
Right Coronary Artery	-0.04874	0.09942	-0.49	0.624	
Thoracic Aorta	0.01421	0.09826	0.145	0.885	**Fatty Streak**
Abdominal Aorta	-0.08715	0.09835	-0.886	0.375585	

Table D. Association between case-control status of raised lesions or fatty streak for each vascular beds and a weighted GRS composed of 19 random SNPs from the 38 SNPs associated with clinical CAD only adjusting for age and sex.

wGRS (=random 19 SNPs from 38 non-risk factors SNPs)	Estimate	SE	Z-value	P	Lesions
Right Coronary Artery	0.10084	0.0993	1.015	0.31	
Thoracic Aorta	0.20327	0.14291	1.422	0.1549	**Raised Lesions**
Abdominal Aorta	0.01097	0.09932	0.11	0.912	
Right Coronary Artery	0.001678	0.10075	0.017	0.987	
Thoracic Aorta	0.11657	0.09897	1.178	0.2389	**Fatty Streak**
Abdominal Aorta	-0.03572	0.09908	-0.361	0.718439	

Table E. Association a weighted GRS of 57 SNPs associated with clinical coronary artery disease and case-control status after further filtering by imputation quality r^2 (0.3; 0.5; 0.8).

GRS (SNPs)		Trait	OR	Lower 95% CI	Higher 95% CI	P	r^2
20	Raised Lesions	Right Coronary Artery	1.34	1.10	1.64	0.004	0.3
		Thoracic Aorta	1.21	0.91	1.59	0.185	
		Abdominal Aorta	1.19	0.98	1.45	0.077	
	Fatty Streaks	Right Coronary Artery	1.34	1.10	1.64	0.004	
		Thoracic Aorta	0.97	0.80	1.18	0.773	
		Abdominal Aorta	0.93	0.76	1.12	0.441	
12	Raised Lesions	Right Coronary Artery	1.31	1.08	1.60	0.007	0.5
		Thoracic Aorta	1.14	0.86	1.50	0.356	
		Abdominal Aorta	1.19	0.98	1.44	0.080	
	Fatty Streaks	Right Coronary Artery	1.31	1.07	1.59	0.008	
		Thoracic Aorta	0.96	0.79	1.16	0.678	
		Abdominal Aorta	0.89	0.73	1.08	0.226	
4	Raised Lesions	Right Coronary Artery	1.34	1.10	1.63	0.004	0.8
		Thoracic Aorta	1.06	0.80	1.41	0.684	
		Abdominal Aorta	1.11	0.91	1.35	0.307	
	Fatty Streaks	Right Coronary Artery	1.11	0.91	1.35	0.300	
		Thoracic Aorta	1.08	0.89	1.32	0.420	
		Abdominal Aorta	0.85	0.70	1.04	0.119	

Table F. Association a weighted GRS restricted to 38 SNPs not associated with traditional risk factors and case-control status after further filtering by imputation quality r^2 (0.3; 0.5; 0.8).

GRS (SNPs)		Trait	OR	Lower 95% CI	Higher 95% CI	P	r^2
15	Raised Lesions	Right Coronary Artery	1.34	1.10	1.64	0.004	0.3
		Thoracic Aorta	1.19	0.90	1.57	0.223	
		Abdominal Aorta	1.17	0.96	1.42	0.121	
	Fatty Streaks	Right Coronary Artery	1.31	1.07	1.60	0.008	
		Thoracic Aorta	0.99	0.81	1.20	0.893	
		Abdominal Aorta	0.91	0.75	1.11	0.358	
9	Raised Lesions	Right Coronary Artery	1.27	1.05	1.55	0.016	0.5
		Thoracic Aorta	1.16	0.88	1.52	0.303	
		Abdominal Aorta	1.16	0.96	1.41	0.133	
	Fatty Streaks	Right Coronary Artery	1.29	1.06	1.58	0.011	
		Thoracic Aorta	0.95	0.78	1.15	0.580	
		Abdominal Aorta	0.88	0.72	1.06	0.186	
3	Raised Lesions	Right Coronary Artery	1.18	0.97	1.43	0.094	0.8
		Thoracic Aorta	1.07	0.81	1.41	0.655	
		Abdominal Aorta	1.04	0.86	1.27	0.659	
	Fatty Streaks	Right Coronary Artery	1.03	0.85	1.26	0.747	
		Thoracic Aorta	0.96	0.79	1.17	0.679	
		Abdominal Aorta	0.76	0.62	0.93	0.008	

Table G. Association between weighted GRS and case-control status when using genotypes imputed with the Haplotype Reference Consortium.

GRS		Trait	OR	Lower 95% CI	Higher 95% CI	P
**Using all 55 imputable SNPs**	**Raised Lesions**	Right Coronary Artery	1.27	1.05	1.55	0.016
		Thoracic Aorta	1.22	0.92	1.61	0.163
		Abdominal Aorta	1.22	1.00	1.49	0.045
	**Fatty Streaks**	Right Coronary Artery	1.28	1.05	1.56	0.016
		Thoracic Aorta	0.97	0.80	1.18	0.764
		Abdominal Aorta	0.98	0.81	1.19	0.828
**Using 36 imputable SNPs not associated with traditional risk factors**	**Raised Lesions**	Right Coronary Artery	1.30	1.07	1.58	0.0094
		Thoracic Aorta	1.20	0.91	1.59	0.1993
		Abdominal Aorta	1.24	1.02	1.50	0.0342
	**Fatty Streaks**	Right Coronary Artery	1.26	1.04	1.54	0.0213
		Thoracic Aorta	0.98	0.81	1.19	0.8296
		Abdominal Aorta	0.97	0.80	1.18	0.7727

Table H. Age and sex adjusted association with case-control status of right coronary raised lesions for each of the 57 single nucleotide polymorphisms used to generate the weighted genetic risk score, ranked by p-value from lowest to highest.

SNP	Chr	Risk Allele	Beta	Gene	Freq1	Rsq	Estimate	SE	pval
rs6544713	2	T	0.061	ABCG5/ABCG8	0.784	0.963	6.573	2.530	0.009
rs2246833	10	T	0.055	LIPA	0.595	0.534	6.615	3.345	0.048
rs11203042	10	T	0.039	LIPA	0.605	0.821	7.102	3.911	0.069
rs515135	2	C	0.075	APOB	0.711	0.302	-6.566	3.965	0.098
rs12190287	6	C	0.072	TCF21	0.713	0.338	5.544	3.465	0.110
rs1333049	9	C	0.207	CDKN2BAS	0.602	0.746	1.119	0.721	0.121
rs3217992	9	T	0.145	CDKN2BAS/MTAP/CDKN2B	0.661	0.131	3.932	2.598	0.130
rs10947789	6	T	0.060	KCNK5	0.804	0.047	-20.173	14.483	0.164
rs501120	10	T	0.067	CXCL12	0.718	0.375	6.106	4.494	0.174
rs9326246	11	C	0.086	ZNF259/APO5A/APOA1	0.894	0.004	-60.265	44.459	0.175
rs2505083	10	C	0.061	KIAA1462	0.693	0.459	4.358	3.271	0.183
rs4773144	13	G	0.068	COL4A1/COL4A2	0.598	0.007	126.616	97.596	0.195
rs17114036	1	A	0.106	PPAP2B	0.904	0.125	7.683	6.616	0.246
rs2047009	10	G	0.053	CXCL12/AX747950	0.651	0.723	3.429	2.999	0.253
rs7173743	15	T	0.065	ADAMTS7/MRG15	0.494	0.203	-5.601	5.131	0.275
rs7212798	17	C	0.077	BCAS3	0.694	0.377	-4.461	4.112	0.278
rs2023938	7	C	0.073	HDAC9	0.888	0.125	11.915	12.923	0.357
rs10840293	11	A	0.058	SWAP70	0.510	0.011	21.694	23.900	0.364
rs9515203	13	T	0.079	COL4A1/COL4A2	0.768	0.214	3.954	4.414	0.370
rs13211739	6	G	0.058	PHACTR1	0.856	0.053	13.611	15.217	0.371
rs9319428	13	A	0.055	FLT1	0.690	0.849	2.703	3.085	0.381
rs2954029	8	A	0.048	TRIB1	0.580	0.165	-8.231	10.144	0.417
rs12205331	6	C	0.042	ANKS1A	0.891	0.064	-18.375	23.255	0.429
rs12936587	17	G	0.055	RAI1/PEMT/RASD1	0.615	0.382	3.313	4.267	0.438
rs974819	11	T	0.065	PDGFD	0.578	0.091	6.320	8.213	0.442
rs15563	17	G	0.037	UBE2Z	0.559	0.055	-17.400	22.709	0.444
rs17087335	4	T	0.058	REST-NOA1	0.782	0.500	2.869	4.089	0.483
rs17514846	15	A	0.058	FES/FURIN	0.525	0.021	18.121	26.119	0.488
rs2351524	2	T	0.115	WDR12/ALS2CR16	0.911	0.074	-4.607	7.035	0.513
rs4252120	6	T	0.062	PLG	0.787	0.123	-4.946	7.623	0.516
rs9982601	21	T	0.119	KCNE2/C21orf82	0.862	0.082	-4.531	7.013	0.518
rs495828	9	T	0.066	ABO	0.816	0.264	3.542	5.495	0.519
rs7692387	4	G	0.065	GUCY1A3	0.830	0.082	6.230	9.932	0.531
rs1561198	2	T	0.052	GGCX/VAMP10/VAMP8	0.469	0.100	5.809	9.440	0.538

rs663129	18	A	0.058	PMAIP1-MC4R	0.732	0.781	-1.610	3.251	0.621
rs1429141	4	T	0.066	EDNRA	0.630	0.048	9.701	20.752	0.640
rs2048327	6	C	0.060	SLC22A3/LPAL2/LPA	0.699	0.048	-4.850	10.469	0.643
rs2895811	14	C	0.056	HHIPL1	0.677	0.158	-2.823	6.624	0.670
rs11072794	15	T	0.066	ADAMTS7/DQ582071	0.537	0.131	-2.273	5.391	0.673
rs1122608	19	G	0.092	LDLR/SMARCA4	0.867	0.009	-10.739	25.530	0.674
rs2306374	3	C	0.073	MRAS	0.904	0.993	1.172	2.928	0.689
rs180803	22	G	0.182	POM121L9P-ADORA2A	0.946	0.009	-8.183	21.255	0.700
rs56062135	15	C	0.068	SMAD3	0.881	0.244	-2.196	5.937	0.711
rs11206510	1	T	0.055	PCSK9	0.886	0.029	-8.665	26.167	0.741
rs8042271	15	G	0.095	MFGE8-ABHD2	0.709	0.013	-8.299	25.178	0.742
rs3184504	12	T	0.068	SH2B3	0.732	0.335	-1.005	3.652	0.783
rs2281727	17	G	0.050	SMG6	0.587	0.754	-0.870	3.229	0.788
rs4845625	1	T	0.049	IL6R	0.583	0.004	11.782	46.963	0.802
rs3918226	7	T	0.131	NOS3	0.956	0.012	-4.800	22.881	0.834
rs264	8	G	0.071	LPL	0.845	0.299	0.794	4.552	0.862
rs2252641	2	C	0.048	ZEB2-AC074093.1	0.593	0.649	-0.522	3.756	0.890
rs445925	19	G	0.119	APOE/APOC1/TOMM40	0.867	0.011	2.923	21.678	0.893
rs11556924	7	C	0.083	ZC3HC1	0.809	0.005	-4.423	41.396	0.915
rs602633	1	G	0.116	PSRC1/SORT1	0.607	0.204	0.305	2.972	0.918
rs11191447	10	C	0.087	CYP17A1/CNNM2/NT5C2	0.917	0.618	-0.310	3.388	0.927
rs273909	5	G	0.077	SLC22A4/SLC22A5	0.936	0.374	0.133	5.511	0.981
rs2075650	19	G	0.106	APOE/APOC1/TOMM40	0.863	0.022	-0.254	12.533	0.984
**Meta-Analysis**	.	.	.	.	.	.	1.434	0.482	0.003

Table I. Age and sex adjusted association with case-control status of right coronary fatty streak for each of the 57 single nucleotide polymorphisms used to generate the weighted genetic risk score, ranked by p-value from lowest to highest.

SNP	Chr	Risk Allele	Beta	Gene	Freq1	Rsq	Estimate	SE	pval
rs3217992	9	T	0.145	CDKN2BAS/MTAP/CDKN2B	0.661	0.131	7.739	2.672	0.004
rs2954029	8	A	0.048	TRIB1	0.580	0.165	-27.128	10.564	0.010
rs17087335	4	T	0.058	REST-NOA1	0.782	0.500	10.210	4.051	0.012
rs1333049	9	C	0.207	CDKN2BAS	0.602	0.746	1.815	0.738	0.014
rs9319428	13	A	0.055	FLT1	0.690	0.849	-6.587	3.267	0.044
rs10947789	6	T	0.060	KCNK5	0.804	0.047	-29.271	14.721	0.047
rs17114036	1	A	0.106	PPAP2B	0.904	0.125	10.172	6.805	0.135
rs2306374	3	C	0.073	MRAS	0.904	0.993	4.286	2.891	0.138
rs6544713	2	T	0.061	ABCG5/ABCG8	0.784	0.963	3.261	2.576	0.206
rs9515203	13	T	0.079	COL4A1/COL4A2	0.768	0.214	5.657	4.504	0.209
rs11203042	10	T	0.039	LIPA	0.605	0.821	4.972	3.969	0.210
rs8042271	15	G	0.095	MFGE8-ABHD2	0.709	0.013	-31.564	25.430	0.215
rs11206510	1	T	0.055	PCSK9	0.886	0.029	-32.086	26.023	0.218
rs1122608	19	G	0.092	LDLR/SMARCA4	0.867	0.009	32.288	26.344	0.220
rs2246833	10	T	0.055	LIPA	0.595	0.534	4.094	3.385	0.227
rs2351524	2	T	0.115	WDR12/ALS2CR16	0.911	0.074	-8.602	7.174	0.231
rs7692387	4	G	0.065	GUCY1A3	0.830	0.082	-11.387	9.628	0.237
rs1429141	4	T	0.066	EDNRA	0.630	0.048	22.519	21.333	0.291
rs3184504	12	T	0.068	SH2B3	0.732	0.335	3.771	3.692	0.307
rs56062135	15	C	0.068	SMAD3	0.881	0.244	5.869	6.164	0.341
rs663129	18	A	0.058	PMAIP1-MC4R	0.732	0.781	-3.090	3.326	0.353
rs4845625	1	T	0.049	IL6R	0.583	0.004	-43.495	47.246	0.357
rs7173743	15	T	0.065	ADAMTS7/MRG15	0.494	0.203	4.709	5.231	0.368
rs12190287	6	C	0.072	TCF21	0.713	0.338	3.111	3.457	0.368
rs11191447	10	C	0.087	CYP17A1/CNNM2/NT5C2	0.917	0.618	3.063	3.574	0.391
rs974819	11	T	0.065	PDGFD	0.578	0.091	-7.225	8.465	0.393
rs1561198	2	T	0.052	GGCX/VAMP10/VAMP8	0.469	0.100	-8.037	9.726	0.409
rs4252120	6	T	0.062	PLG	0.787	0.123	-6.232	7.714	0.419
rs2048327	6	C	0.060	SLC22A3/LPAL2/LPA	0.699	0.048	8.195	10.572	0.438
rs15563	17	G	0.037	UBE2Z	0.559	0.055	-16.996	23.103	0.462
rs4773144	13	G	0.068	COL4A1/COL4A2	0.598	0.007	57.841	86.332	0.503
rs602633	1	G	0.116	PSRC1/SORT1	0.607	0.204	1.983	3.026	0.512
rs12936587	17	G	0.055	RAI1/PEMT/RASD1	0.615	0.382	2.551	4.335	0.556
rs2075650	19	G	0.106	APOE/APOC1/TOMM40	0.863	0.022	-7.125	12.775	0.577

rs273909	5	G	0.077	SLC22A4/SLC22A5	0.936	0.374	-3.146	5.735	0.583
rs12205331	6	C	0.042	ANKS1A	0.891	0.064	-11.747	23.502	0.617
rs10840293	11	A	0.058	SWAP70	0.510	0.011	10.640	24.031	0.658
rs2047009	10	G	0.053	CXCL12/AX747950	0.651	0.723	-1.234	3.035	0.684
rs11072794	15	T	0.066	ADAMTS7/DQ582071	0.537	0.131	-2.104	5.447	0.699
rs180803	22	G	0.182	POM121L9P-ADORA2A	0.946	0.009	-5.534	21.445	0.796
rs2281727	17	G	0.050	SMG6	0.587	0.754	-0.828	3.275	0.800
rs3918226	7	T	0.131	NOS3	0.956	0.012	-5.471	23.167	0.813
rs501120	10	T	0.067	CXCL12	0.718	0.375	0.976	4.329	0.822
rs445925	19	G	0.119	APOE/APOC1/TOMM40	0.867	0.011	-4.526	21.790	0.835
rs11556924	7	C	0.083	ZC3HC1	0.809	0.005	-8.567	42.355	0.840
rs2895811	14	C	0.056	HHIPL1	0.677	0.158	-1.309	6.716	0.845
rs495828	9	T	0.066	ABO	0.816	0.264	1.040	5.588	0.852
rs9326246	11	C	0.086	ZNF259/APO5A/APOA1	0.894	0.004	7.942	42.790	0.853
rs13211739	6	G	0.058	PHACTR1	0.856	0.053	-2.536	15.689	0.872
rs7212798	17	C	0.077	BCAS3	0.694	0.377	0.605	3.990	0.879
rs2252641	2	C	0.048	ZEB2-AC074093.1	0.593	0.649	-0.553	3.802	0.884
rs2505083	10	C	0.061	KIAA1462	0.693	0.459	-0.484	3.329	0.884
rs2023938	7	C	0.073	HDAC9	0.888	0.125	1.339	13.439	0.921
rs9982601	21	T	0.119	KCNE2/C21orf82	0.862	0.082	-0.253	7.052	0.971
rs17514846	15	A	0.058	FES/FURIN	0.525	0.021	0.585	26.976	0.983
rs515135	2	C	0.075	APOB	0.711	0.302	0.069	4.127	0.987
rs264	8	G	0.071	LPL	0.845	0.299	0.072	4.566	0.987
**Meta-Analysis**	.	.	.	.	.	.	1.587	0.486	0.001

Table J. Age and sex adjusted association with case-control status of thoracic aorta raised lesions for each of the 57 single nucleotide polymorphisms used to generate the weighted genetic risk score, ranked by p-value from lowest to highest.

SNP	Chr	Risk Allele	Beta	Gene	Freq1	Rsq	Estimate	SE	pval
rs495828	9	T	0.066	ABO	0.816	0.264	21.908	7.832	0.005
rs2048327	6	C	0.060	SLC22A3/LPAL2/LPA	0.699	0.048	-35.752	15.213	0.019
rs17087335	4	T	0.058	REST-NOA1	0.782	0.500	11.152	5.435	0.040
rs180803	22	G	0.182	POM121L9P-ADORA2A	0.946	0.009	-66.064	33.434	0.048
rs13211739	6	G	0.058	PHACTR1	0.856	0.053	40.585	20.613	0.049
rs1561198	2	T	0.052	GGCX/VAMP10/VAMP8	0.469	0.100	25.353	13.537	0.061
rs56062135	15	C	0.068	SMAD3	0.881	0.244	17.594	9.518	0.065
rs12205331	6	C	0.042	ANKS1A	0.891	0.064	56.334	32.345	0.082
rs445925	19	G	0.119	APOE/APOC1/TOMM40	0.867	0.011	59.376	34.908	0.089
rs2351524	2	T	0.115	WDR12/ALS2CR16	0.911	0.074	15.415	9.661	0.111
rs11072794	15	T	0.066	ADAMTS7/DQ582071	0.537	0.131	-11.524	7.449	0.122
rs10947789	6	T	0.060	KCNK5	0.804	0.047	-30.177	20.668	0.144
rs17514846	15	A	0.058	FES/FURIN	0.525	0.021	-59.319	41.890	0.157
rs11556924	7	C	0.083	ZC3HC1	0.809	0.005	-92.760	65.594	0.157
rs4252120	6	T	0.062	PLG	0.787	0.123	-15.105	10.873	0.165
rs9326246	11	C	0.086	ZNF259/APO5A/APOA1	0.894	0.004	-80.317	66.694	0.228
rs9982601	21	T	0.119	KCNE2/C21orf82	0.862	0.082	11.773	9.841	0.232
rs17114036	1	A	0.106	PPAP2B	0.904	0.125	10.908	10.020	0.276
rs11206510	1	T	0.055	PCSK9	0.886	0.029	-38.767	35.765	0.278
rs3217992	9	T	0.145	CDKN2BAS/MTAP/CDKN2B	0.661	0.131	4.027	3.728	0.280
rs9515203	13	T	0.079	COL4A1/COL4A2	0.768	0.214	-6.227	5.794	0.282
rs1333049	9	C	0.207	CDKN2BAS	0.602	0.746	1.103	1.035	0.287
rs273909	5	G	0.077	SLC22A4/SLC22A5	0.936	0.374	7.708	7.341	0.294
rs663129	18	A	0.058	PMAIP1-MC4R	0.732	0.781	-4.853	4.869	0.319
rs2252641	2	C	0.048	ZEB2-AC074093.1	0.593	0.649	-5.365	5.395	0.320
rs12936587	17	G	0.055	RAI1/PEMT/RASD1	0.615	0.382	5.870	6.220	0.345
rs515135	2	C	0.075	APOB	0.711	0.302	5.664	6.142	0.356
rs10840293	11	A	0.058	SWAP70	0.510	0.011	-29.832	33.440	0.372
rs1122608	19	G	0.092	LDLR/SMARCA4	0.867	0.009	-31.870	36.388	0.381
rs3918226	7	T	0.131	NOS3	0.956	0.012	27.593	32.128	0.390
rs7173743	15	T	0.065	ADAMTS7/MRG15	0.494	0.203	-6.262	7.304	0.391
rs7692387	4	G	0.065	GUCY1A3	0.830	0.082	11.514	14.698	0.433
rs3184504	12	T	0.068	SH2B3	0.732	0.335	3.942	5.193	0.448
rs9319428	13	A	0.055	FLT1	0.690	0.849	3.022	4.410	0.493

rs602633	1	G	0.116	PSRC1/SORT1	0.607	0.204	2.922	4.312	0.498
rs2505083	10	C	0.061	KIAA1462	0.693	0.459	-3.206	4.741	0.499
rs15563	17	G	0.037	UBE2Z	0.559	0.055	22.747	34.225	0.506
rs11191447	10	C	0.087	CYP17A1/CNNM2/NT5C2	0.917	0.618	3.299	5.113	0.519
rs974819	11	T	0.065	PDGFD	0.578	0.091	7.489	11.629	0.520
rs11203042	10	T	0.039	LIPA	0.605	0.821	-3.605	5.758	0.531
rs2047009	10	G	0.053	CXCL12/AX747950	0.651	0.723	-2.702	4.319	0.532
rs501120	10	T	0.067	CXCL12	0.718	0.375	3.816	6.436	0.553
rs2075650	19	G	0.106	APOE/APOC1/TOMM40	0.863	0.022	-10.078	18.225	0.580
rs8042271	15	G	0.095	MFGE8-ABHD2	0.709	0.013	-19.468	36.117	0.590
rs2306374	3	C	0.073	MRAS	0.904	0.993	2.023	4.077	0.620
rs12190287	6	C	0.072	TCF21	0.713	0.338	1.585	4.792	0.741
rs2954029	8	A	0.048	TRIB1	0.580	0.165	-3.325	14.562	0.819
rs4773144	13	G	0.068	COL4A1/COL4A2	0.598	0.007	-22.122	99.238	0.824
rs1429141	4	T	0.066	EDNRA	0.630	0.048	6.421	29.626	0.828
rs2023938	7	C	0.073	HDAC9	0.888	0.125	3.525	18.650	0.850
rs4845625	1	T	0.049	IL6R	0.583	0.004	11.757	66.847	0.860
rs2895811	14	C	0.056	HHIPL1	0.677	0.158	1.100	9.466	0.907
rs7212798	17	C	0.077	BCAS3	0.694	0.377	-0.667	5.751	0.908
rs6544713	2	T	0.061	ABCG5/ABCG8	0.784	0.963	0.263	3.678	0.943
rs2246833	10	T	0.055	LIPA	0.595	0.534	0.166	4.810	0.973
rs2281727	17	G	0.050	SMG6	0.587	0.754	0.023	4.601	0.996
rs264	8	G	0.071	LPL	0.845	0.299	0.011	6.366	0.999
**Meta-Analysis**	.	.	.	.	.	.	1.259	0.69	0.068

Table K. Age and sex adjusted association with case-control status of thoracic aorta fatty streak for each of the 57 single nucleotide polymorphisms used to generate the weighted genetic risk score, ranked by p-value from lowest to highest.

SNP	Chr	Risk.Allele	Beta	Gene	Freq1	Rsq	Estimate	SE	pval
rs3184504	12	T	0.068	SH2B3	0.732	0.335	-9.399	3.734	0.012
rs1122608	19	G	0.092	LDLR/SMARCA4	0.867	0.009	57.251	26.406	0.030
rs3918226	7	T	0.131	NOS3	0.956	0.012	-48.948	23.643	0.038
rs11191447	10	C	0.087	CYP17A1/CNNM2/NT5C2	0.917	0.618	-6.559	3.190	0.040
rs6544713	2	T	0.061	ABCG5/ABCG8	0.784	0.963	4.857	2.525	0.054
rs602633	1	G	0.116	PSRC1/SORT1	0.607	0.204	5.187	3.057	0.090
rs17087335	4	T	0.058	REST-NOA1	0.782	0.500	-7.174	4.410	0.104
rs4845625	1	T	0.049	IL6R	0.583	0.004	71.448	47.563	0.133
rs2246833	10	T	0.055	LIPA	0.595	0.534	4.830	3.315	0.145
rs2954029	8	A	0.048	TRIB1	0.580	0.165	-13.876	10.151	0.172
rs11072794	15	T	0.066	ADAMTS7/DQ582071	0.537	0.131	-7.062	5.353	0.187
rs2252641	2	C	0.048	ZEB2-AC074093.1	0.593	0.649	-4.832	3.774	0.200
rs10840293	11	A	0.058	SWAP70	0.510	0.011	-29.135	23.765	0.220
rs8042271	15	G	0.095	MFGE8-ABHD2	0.709	0.013	-30.462	24.913	0.221
rs56062135	15	C	0.068	SMAD3	0.881	0.244	7.272	6.131	0.236
rs11206510	1	T	0.055	PCSK9	0.886	0.029	-30.441	25.699	0.236
rs17514846	15	A	0.058	FES/FURIN	0.525	0.021	-32.724	27.646	0.237
rs11556924	7	C	0.083	ZC3HC1	0.809	0.005	46.823	39.703	0.238
rs2306374	3	C	0.073	MRAS	0.904	0.993	-3.674	3.140	0.242
rs13211739	6	G	0.058	PHACTR1	0.856	0.053	17.611	15.072	0.243
rs11203042	10	T	0.039	LIPA	0.605	0.821	4.346	3.888	0.264
rs9515203	13	T	0.079	COL4A1/COL4A2	0.768	0.214	4.815	4.447	0.279
rs7212798	17	C	0.077	BCAS3	0.694	0.377	3.997	3.816	0.295
rs501120	10	T	0.067	CXCL12	0.718	0.375	4.552	4.408	0.302
rs7173743	15	T	0.065	ADAMTS7/MRG15	0.494	0.203	4.991	5.157	0.333
rs264	8	G	0.071	LPL	0.845	0.299	4.558	4.721	0.334
rs2047009	10	G	0.053	CXCL12/AX747950	0.651	0.723	2.778	2.983	0.352
rs495828	9	T	0.066	ABO	0.816	0.264	-4.858	5.568	0.383
rs1429141	4	T	0.066	EDNRA	0.630	0.048	-17.403	20.154	0.388
rs515135	2	C	0.075	APOB	0.711	0.302	3.360	4.161	0.419
rs12190287	6	C	0.072	TCF21	0.713	0.338	2.548	3.419	0.456
rs2281727	17	G	0.050	SMG6	0.587	0.754	-2.383	3.235	0.461
rs273909	5	G	0.077	SLC22A4/SLC22A5	0.936	0.374	3.666	5.346	0.493
rs17114036	1	A	0.106	PPAP2B	0.904	0.125	4.431	6.467	0.493

rs2351524	2	T	0.115	WDR12/ALS2CR16	0.911	0.074	-4.582	7.024	0.514
rs9326246	11	C	0.086	ZNF259/APO5A/APOA1	0.894	0.004	24.537	41.446	0.554
rs974819	11	T	0.065	PDGFD	0.578	0.091	3.723	8.232	0.651
rs2895811	14	C	0.056	HHIPL1	0.677	0.158	2.830	6.653	0.671
rs9982601	21	T	0.119	KCNE2/C21orf82	0.862	0.082	-2.910	6.952	0.676
rs4773144	13	G	0.068	COL4A1/COL4A2	0.598	0.007	33.285	86.373	0.700
rs12936587	17	G	0.055	RAI1/PEMT/RASD1	0.615	0.382	-1.580	4.206	0.707
rs9319428	13	A	0.055	FLT1	0.690	0.849	-1.118	3.125	0.720
rs1333049	9	C	0.207	CDKN2BAS	0.602	0.746	-0.255	0.715	0.722
rs3217992	9	T	0.145	CDKN2BAS/MTAP/CDKN2B	0.661	0.131	-0.795	2.582	0.758
rs1561198	2	T	0.052	GGCX/VAMP10/VAMP8	0.469	0.100	-2.590	9.461	0.784
rs12205331	6	C	0.042	ANKS1A	0.891	0.064	-5.843	22.901	0.799
rs2505083	10	C	0.061	KIAA1462	0.693	0.459	-0.759	3.304	0.818
rs180803	22	G	0.182	POM121L9P-ADORA2A	0.946	0.009	-4.355	21.151	0.837
rs663129	18	A	0.058	PMAIP1-MC4R	0.732	0.781	0.512	3.198	0.873
rs2048327	6	C	0.060	SLC22A3/LPAL2/LPA	0.699	0.048	-1.581	10.459	0.880
rs2023938	7	C	0.073	HDAC9	0.888	0.125	1.760	13.235	0.894
rs10947789	6	T	0.060	KCNK5	0.804	0.047	-1.266	14.419	0.930
rs4252120	6	T	0.062	PLG	0.787	0.123	-0.648	7.619	0.932
rs7692387	4	G	0.065	GUCY1A3	0.830	0.082	0.655	9.740	0.946
rs15563	17	G	0.037	UBE2Z	0.559	0.055	1.360	22.831	0.953
rs445925	19	G	0.119	APOE/APOC1/TOMM40	0.867	0.011	-0.833	21.518	0.969
rs2075650	19	G	0.106	APOE/APOC1/TOMM40	0.863	0.022	0.175	12.480	0.989
**Meta-Analysis**	.	.	.	.	.	.	0.007	0.483	0.988

Table L. Age and sex adjusted association with case-control status of abdominal aorta raised lesions for each of the 57 single nucleotide polymorphisms used to generate the weighted genetic risk score, ranked by p-value from lowest to highest.

SNP	Chr	Risk.Allele	Beta	Gene	Freq1	Rsq	Estimate	SE	pval
rs2351524	2	T	0.115	WDR12/ALS2CR16	0.911	0.074	-18.351	7.270	0.012
rs9326246	11	C	0.086	ZNF259/APO5A/APOA1	0.894	0.004	-103.958	46.316	0.025
rs1333049	9	C	0.207	CDKN2BAS	0.602	0.746	1.463	0.724	0.043
rs3217992	9	T	0.145	CDKN2BAS/MTAP/CDKN2B	0.661	0.131	5.064	2.605	0.052
rs1429141	4	T	0.066	EDNRA	0.630	0.048	40.871	21.689	0.060
rs17114036	1	A	0.106	PPAP2B	0.904	0.125	12.888	6.915	0.062
rs2047009	10	G	0.053	CXCL12/AX747950	0.651	0.723	-4.297	3.007	0.153
rs2075650	19	G	0.106	APOE/APOC1/TOMM40	0.863	0.022	16.924	12.445	0.174
rs974819	11	T	0.065	PDGFD	0.578	0.091	11.003	8.180	0.179
rs12205331	6	C	0.042	ANKS1A	0.891	0.064	-29.971	23.479	0.202
rs17514846	15	A	0.058	FES/FURIN	0.525	0.021	-32.787	27.778	0.238
rs13211739	6	G	0.058	PHACTR1	0.856	0.053	17.554	15.186	0.248
rs10947789	6	T	0.060	KCNK5	0.804	0.047	-16.082	14.457	0.266
rs11191447	10	C	0.087	CYP17A1/CNNM2/NT5C2	0.917	0.618	3.952	3.575	0.269
rs6544713	2	T	0.061	ABCG5/ABCG8	0.784	0.963	2.787	2.545	0.274
rs1122608	19	G	0.092	LDLR/SMARCA4	0.867	0.009	-26.123	25.446	0.305
rs11072794	15	T	0.066	ADAMTS7/DQ582071	0.537	0.131	-5.411	5.361	0.313
rs56062135	15	C	0.068	SMAD3	0.881	0.244	5.744	6.088	0.345
rs501120	10	T	0.067	CXCL12	0.718	0.375	4.062	4.393	0.355
rs9515203	13	T	0.079	COL4A1/COL4A2	0.768	0.214	4.017	4.409	0.362
rs2505083	10	C	0.061	KIAA1462	0.693	0.459	2.869	3.272	0.381
rs180803	22	G	0.182	POM121L9P-ADORA2A	0.946	0.009	-18.676	21.461	0.384
rs7692387	4	G	0.065	GUCY1A3	0.830	0.082	-7.805	9.556	0.414
rs15563	17	G	0.037	UBE2Z	0.559	0.055	-18.440	22.700	0.417
rs2048327	6	C	0.060	SLC22A3/LPAL2/LPA	0.699	0.048	-7.796	10.471	0.457
rs4252120	6	T	0.062	PLG	0.787	0.123	5.478	7.664	0.475
rs445925	19	G	0.119	APOE/APOC1/TOMM40	0.867	0.011	-12.758	21.324	0.550
rs2023938	7	C	0.073	HDAC9	0.888	0.125	-7.960	13.670	0.560
rs1561198	2	T	0.052	GGCX/VAMP10/VAMP8	0.469	0.100	5.387	9.441	0.568
rs7173743	15	T	0.065	ADAMTS7/MRG15	0.494	0.203	-2.869	5.127	0.576
rs2954029	8	A	0.048	TRIB1	0.580	0.165	-5.250	10.113	0.604
rs515135	2	C	0.075	APOB	0.711	0.302	2.092	4.124	0.612
rs3184504	12	T	0.068	SH2B3	0.732	0.335	-1.799	3.654	0.623
rs12190287	6	C	0.072	TCF21	0.713	0.338	-1.576	3.370	0.640

rs663129	18	A	0.058	PMAIP1-MC4R	0.732	0.781	-1.484	3.246	0.647
rs11556924	7	C	0.083	ZC3HC1	0.809	0.005	-18.821	41.825	0.653
rs264	8	G	0.071	LPL	0.845	0.299	1.851	4.584	0.686
rs2895811	14	C	0.056	HHIPL1	0.677	0.158	-2.580	6.621	0.697
rs4773144	13	G	0.068	COL4A1/COL4A2	0.598	0.007	-29.138	78.746	0.711
rs2252641	2	C	0.048	ZEB2-AC074093.1	0.593	0.649	-1.353	3.756	0.719
rs2306374	3	C	0.073	MRAS	0.904	0.993	1.022	2.932	0.727
rs8042271	15	G	0.095	MFGE8-ABHD2	0.709	0.013	8.549	25.346	0.736
rs3918226	7	T	0.131	NOS3	0.956	0.012	-6.939	22.895	0.762
rs11203042	10	T	0.039	LIPA	0.605	0.821	1.098	3.919	0.779
rs9982601	21	T	0.119	KCNE2/C21orf82	0.862	0.082	-1.809	6.968	0.795
rs7212798	17	C	0.077	BCAS3	0.694	0.377	-1.004	3.981	0.801
rs12936587	17	G	0.055	RAI1/PEMT/RASD1	0.615	0.382	-0.980	4.241	0.817
rs17087335	4	T	0.058	REST-NOA1	0.782	0.500	-0.881	4.180	0.833
rs11206510	1	T	0.055	PCSK9	0.886	0.029	-5.336	26.222	0.839
rs10840293	11	A	0.058	SWAP70	0.510	0.011	4.795	23.745	0.840
rs2281727	17	G	0.050	SMG6	0.587	0.754	0.627	3.213	0.845
rs2246833	10	T	0.055	LIPA	0.595	0.534	0.613	3.331	0.854
rs273909	5	G	0.077	SLC22A4/SLC22A5	0.936	0.374	-0.967	5.555	0.862
rs4845625	1	T	0.049	IL6R	0.583	0.004	6.503	46.882	0.890
rs9319428	13	A	0.055	FLT1	0.690	0.849	0.399	3.105	0.898
rs602633	1	G	0.116	PSRC1/SORT1	0.607	0.204	0.371	2.970	0.900
rs495828	9	T	0.066	ABO	0.816	0.264	0.587	5.516	0.915
**Meta-Analysis**	.	.	.	.	.	.	1.01	0.483	0.036

Table M. Age and sex adjusted association with case-control status of abdominal aorta fatty streak for each of the 57 single nucleotide polymorphisms used to generate the weighted genetic risk score, ranked by p-value from lowest to highest.

SNP	Chr	Risk.Allele	Beta	Gene	Freq1	Rsq	Estimate	SE	pval
rs602633	1	G	0.116	PSRC1/SORT1	0.607	0.204	7.832	3.105	0.012
rs12190287	6	C	0.072	TCF21	0.713	0.338	8.198	3.503	0.019
rs2023938	7	C	0.073	HDAC9	0.888	0.125	-33.658	15.110	0.026
rs15563	17	G	0.037	UBE2Z	0.559	0.055	52.355	24.079	0.030
rs11203042	10	T	0.039	LIPA	0.605	0.821	-8.169	4.003	0.041
rs4773144	13	G	0.068	COL4A1/COL4A2	0.598	0.007	218.057	108.143	0.044
rs10840293	11	A	0.058	SWAP70	0.510	0.011	-45.341	23.770	0.056
rs56062135	15	C	0.068	SMAD3	0.881	0.244	11.624	6.226	0.062
rs2246833	10	T	0.055	LIPA	0.595	0.534	-6.061	3.360	0.071
rs2306374	3	C	0.073	MRAS	0.904	0.993	-5.791	3.239	0.074
rs2047009	10	G	0.053	CXCL12/AX747950	0.651	0.723	5.036	2.993	0.092
rs2281727	17	G	0.050	SMG6	0.587	0.754	-5.071	3.273	0.121
rs180803	22	G	0.182	POM121L9P-ADORA2A	0.946	0.009	-33.601	21.759	0.123
rs2954029	8	A	0.048	TRIB1	0.580	0.165	-15.516	10.170	0.127
rs12936587	17	G	0.055	RAI1/PEMT/RASD1	0.615	0.382	5.905	4.265	0.166
rs974819	11	T	0.065	PDGFD	0.578	0.091	9.960	8.154	0.222
rs7212798	17	C	0.077	BCAS3	0.694	0.377	-4.598	4.094	0.261
rs501120	10	T	0.067	CXCL12	0.718	0.375	4.675	4.400	0.288
rs17514846	15	A	0.058	FES/FURIN	0.525	0.021	-29.037	27.477	0.291
rs11072794	15	T	0.066	ADAMTS7/DQ582071	0.537	0.131	5.787	5.480	0.291
rs4845625	1	T	0.049	IL6R	0.583	0.004	49.192	47.067	0.296
rs9982601	21	T	0.119	KCNE2/C21orf82	0.862	0.082	-7.336	7.024	0.296
rs6544713	2	T	0.061	ABCG5/ABCG8	0.784	0.963	2.577	2.536	0.310
rs495828	9	T	0.066	ABO	0.816	0.264	5.412	5.455	0.321
rs1122608	19	G	0.092	LDLR/SMARCA4	0.867	0.009	23.695	25.749	0.357
rs9319428	13	A	0.055	FLT1	0.690	0.849	-2.613	3.137	0.405
rs2048327	6	C	0.060	SLC22A3/LPAL2/LPA	0.699	0.048	-8.496	10.438	0.416
rs1561198	2	T	0.052	GGCX/VAMP10/VAMP8	0.469	0.100	-7.717	9.518	0.418
rs273909	5	G	0.077	SLC22A4/SLC22A5	0.936	0.374	-4.456	5.692	0.434
rs17114036	1	A	0.106	PPAP2B	0.904	0.125	4.893	6.444	0.448
rs2505083	10	C	0.061	KIAA1462	0.693	0.459	-2.475	3.306	0.454
rs663129	18	A	0.058	PMAIP1-MC4R	0.732	0.781	-2.370	3.254	0.466
rs10947789	6	T	0.060	KCNK5	0.804	0.047	10.471	14.426	0.468
rs4252120	6	T	0.062	PLG	0.787	0.123	5.307	7.631	0.487

rs1429141	4	T	0.066	EDNRA	0.630	0.048	13.996	20.764	0.500
rs264	8	G	0.071	LPL	0.845	0.299	-2.653	4.422	0.549
rs13211739	6	G	0.058	PHACTR1	0.856	0.053	-9.179	15.536	0.555
rs11556924	7	C	0.083	ZC3HC1	0.809	0.005	-23.382	41.640	0.574
rs7692387	4	G	0.065	GUCY1A3	0.830	0.082	-5.347	9.561	0.576
rs12205331	6	C	0.042	ANKS1A	0.891	0.064	-12.661	23.015	0.582
rs8042271	15	G	0.095	MFGE8-ABHD2	0.709	0.013	-12.069	25.011	0.629
rs9326246	11	C	0.086	ZNF259/APO5A/APOA1	0.894	0.004	-20.431	42.738	0.633
rs2895811	14	C	0.056	HHIPL1	0.677	0.158	-2.740	6.590	0.678
rs515135	2	C	0.075	APOB	0.711	0.302	1.513	4.093	0.712
rs1333049	9	C	0.207	CDKN2BAS	0.602	0.746	-0.263	0.714	0.713
rs2252641	2	C	0.048	ZEB2-AC074093.1	0.593	0.649	1.312	3.736	0.725
rs11191447	10	C	0.087	CYP17A1/CNNM2/NT5C2	0.917	0.618	-1.151	3.338	0.730
rs2351524	2	T	0.115	WDR12/ALS2CR16	0.911	0.074	-2.174	6.969	0.755
rs2075650	19	G	0.106	APOE/APOC1/TOMM40	0.863	0.022	3.052	12.438	0.806
rs9515203	13	T	0.079	COL4A1/COL4A2	0.768	0.214	0.927	4.314	0.830
rs7173743	15	T	0.065	ADAMTS7/MRG15	0.494	0.203	-0.883	5.107	0.863
rs3918226	7	T	0.131	NOS3	0.956	0.012	3.687	22.670	0.871
rs17087335	4	T	0.058	REST-NOA1	0.782	0.500	0.622	4.122	0.880
rs3217992	9	T	0.145	CDKN2BAS/MTAP/CDKN2B	0.661	0.131	-0.294	2.576	0.909
rs11206510	1	T	0.055	PCSK9	0.886	0.029	1.409	26.303	0.957
rs3184504	12	T	0.068	SH2B3	0.732	0.335	0.092	3.628	0.980
rs445925	19	G	0.119	APOE/APOC1/TOMM40	0.867	0.011	0.489	21.506	0.982
**Meta-Analysis**	.	.	.	.	.	.	-0.144	0.484	0.766

Fig A. Ward's hierarchical clustering analysis (using JMP Genomics SAS) of six phenotypes in all white participants of the Pathobiological Determinants of the Youth study (n=1344) including percent surface area of involvement by raised lesions and fatty streaks in the coronary (CR and TF), thoracic aorta (TR and TF), and abdominal aorta (AR and TF). The algorithms identified 2 clusters. The first cluster includes CF, CR, TR, and AR and the second cluster includes TF and AF. The clusters identified are the same as those identified in the subset of 564 subjects selected for genotyping b SEA investigators.

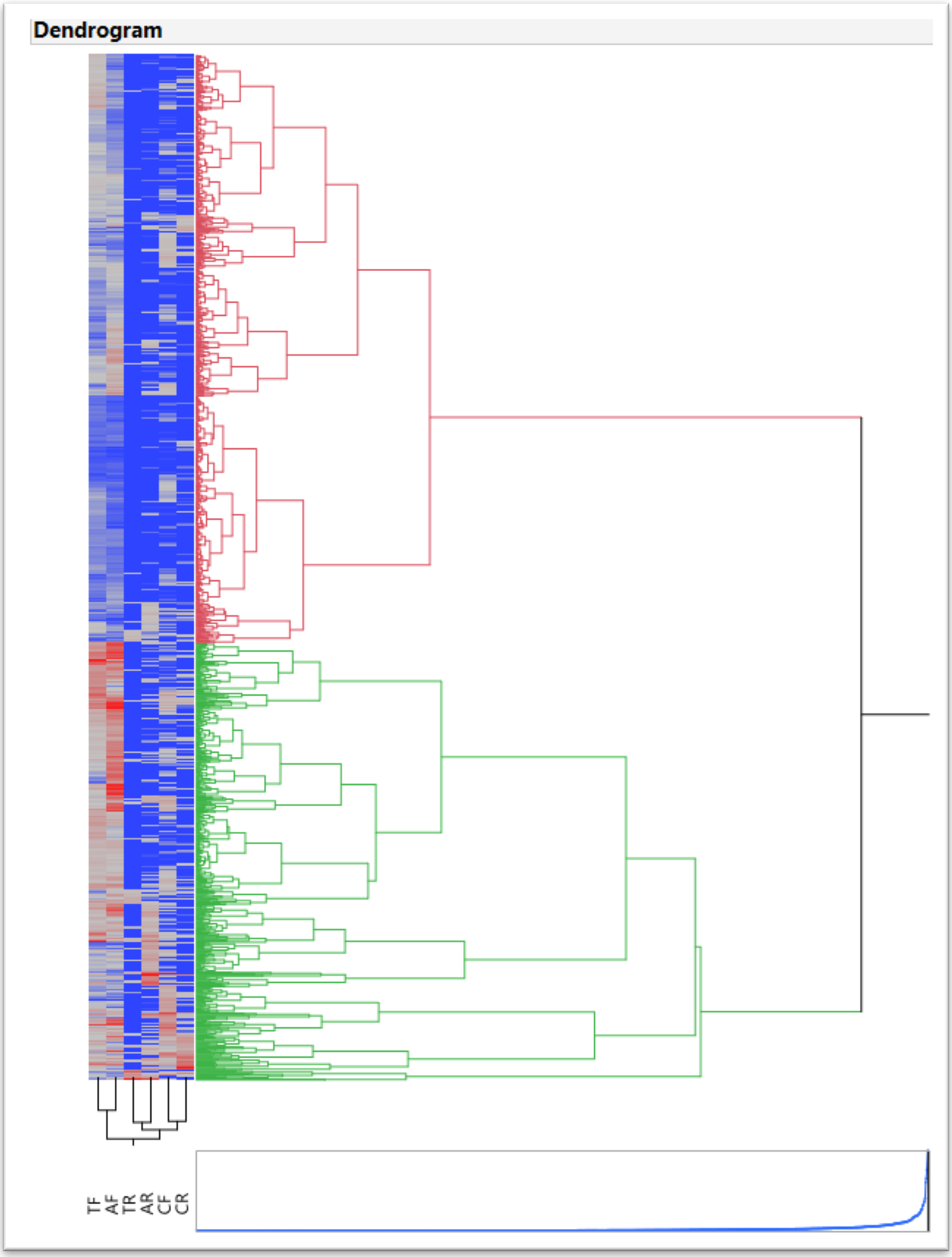

Fig B. Comparison of the quality of imputation between 1000 genomes reference panel (red) and the Haplotype Reference Consortium panel (blue) for each of the 57 SNPs used to construct our weighted genetic risk scores. The y-axis plots the estimated imputation accuracy (r2). The x-axis lists the SNPs in order of increasing imputation r2 when using the 1000 genomes reference panel. Two SNPs could not be imputed using the Haplotype Reference Consortium panel.

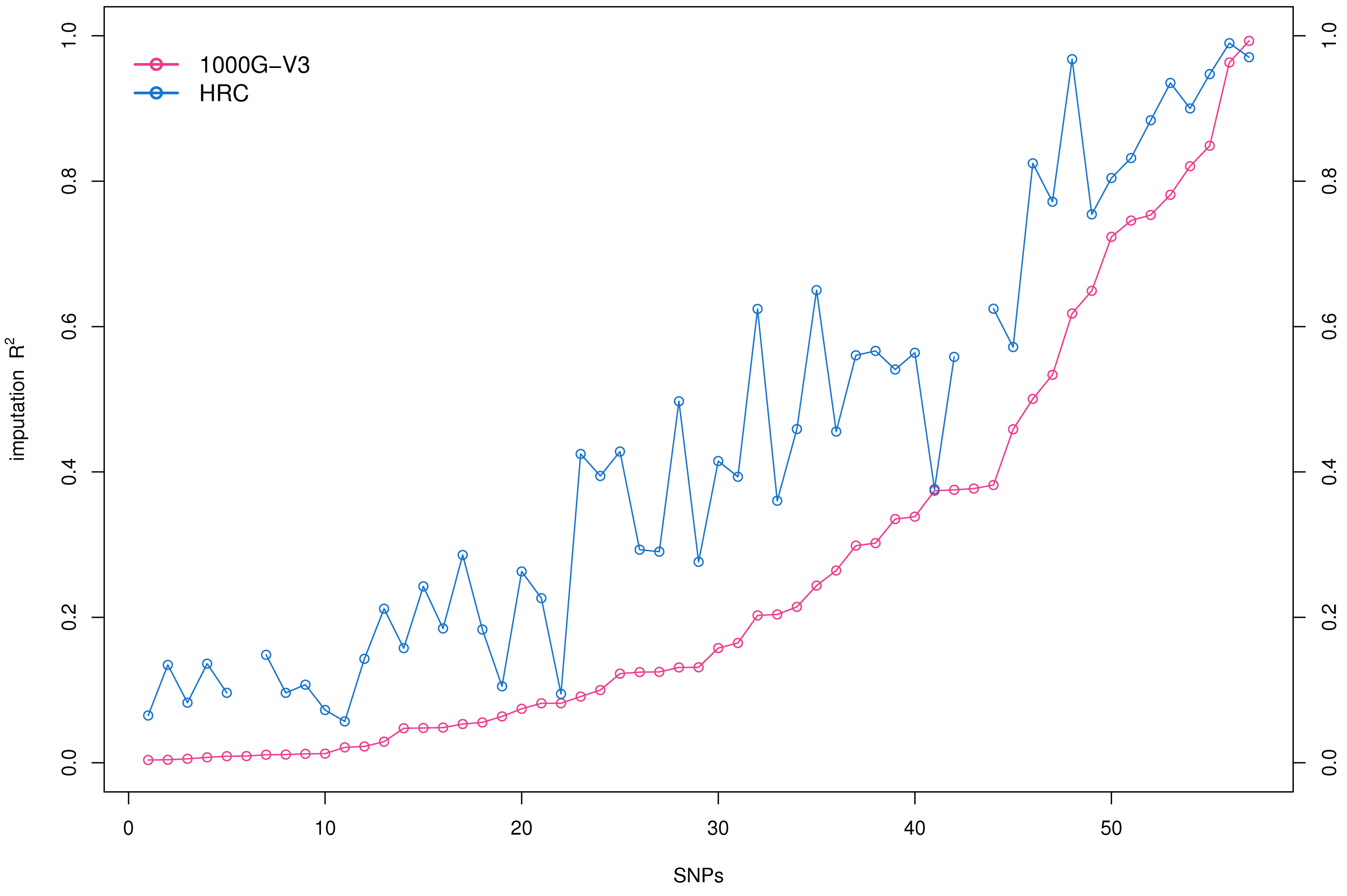

Fig C. Odds Ratio per standard deviation increases in the weighted GRS (wGRS) for being in the top quartile of percent surface area of involvement (case) of early arterial lesions compared to the bottom three quartiles (controls). Odds Ratios are adjusted for age and sex at time of autopsy. Results for all 3 vascular beds are grouped by lesion type (fatty streak vs. raised lesions) and by age group (young 15-27yo vs. old 28-35yo) and shown for wGRS that includes all 57 SNPs associated with clinical CAD.

Study or Subgroup	log[Odds Ratio]	SE	Odds Ratio IV, Fixed, 95% CI	p
**Fatty Streaks (15–27)**				
Right Coronary Artery	0.23	0.17	1.26 [0.90, 1.76]	0.17
Thoracic Aorta	−0.18	0.13	0.84 [0.65, 1.08]	0.24
Abdominal Aorta	−0.05	0.15	0.95 [0.71, 1.28]	0.69
**Subtotal (95% CI)**			**0.97 [0.82, 1.14]**	
Heterogeneity: $Chi^2 = 3.68$, df = 2 (P = 0.16); $I^2 = 46\%$				
Test for overall effect: Z = 0.42 (P = 0.68)				
**Fatty Streaks (28–35)**				
Right Coronary Artery	0.31	0.13	1.36 [1.06, 1.76]	0.01
Thoracic Aorta	0.11	0.13	1.12 [0.87, 1.44]	0.02
Abdominal Aorta	0	0.13	1.00 [0.78, 1.29]	0.99
**Subtotal (95% CI)**			**1.15 [0.99, 1.33]**	
Heterogeneity: $Chi^2 = 2.92$, df = 2 (P = 0.23); $I^2 = 32\%$				
Test for overall effect: Z = 1.87 (P = 0.06)				
**Raised Lesions (15–27)**				
Right Coronary Artery	0.13	0.16	1.14 [0.83, 1.56]	0.39
Thoracic Aorta	0.05	0.24	1.05 [0.66, 1.68]	0.83
Abdominal Aorta	0.02	0.16	1.02 [0.75, 1.40]	0.89
**Subtotal (95% CI)**			**1.07 [0.88, 1.31]**	
Heterogeneity: $Chi^2 = 0.25$, df = 2 (P = 0.88); $I^2 = 0\%$				
Test for overall effect: Z = 0.69 (P = 0.49)				
**Raised Lesions (28–35)**				
Right Coronary Artery	0.32	0.13	1.38 [1.07, 1.78]	0.01
Thoracic Aorta	0.23	0.2	1.26 [0.85, 1.86]	0.04
Abdominal Aorta	0.24	0.13	1.27 [0.99, 1.64]	0.06
**Subtotal (95% CI)**			**1.31 [1.11, 1.54]**	
Heterogeneity: $Chi^2 = 0.24$, df = 2 (P = 0.89); $I^2 = 0\%$				
Test for overall effect: Z = 3.25 (P = 0.001)				
**Total (95% CI)**			**100.0% 1.13 [1.04, 1.22]**	
Heterogeneity: $Chi^2 = 14.02$, df = 11 (P = 0.23); $I^2 = 22\%$				
Test for overall effect: Z = 2.78 (P = 0.005)				
Test for subgroup differences: $Chi^2 = 6.93$, df = 3 (P = 0.07), $I^2 = 56.7\%$				

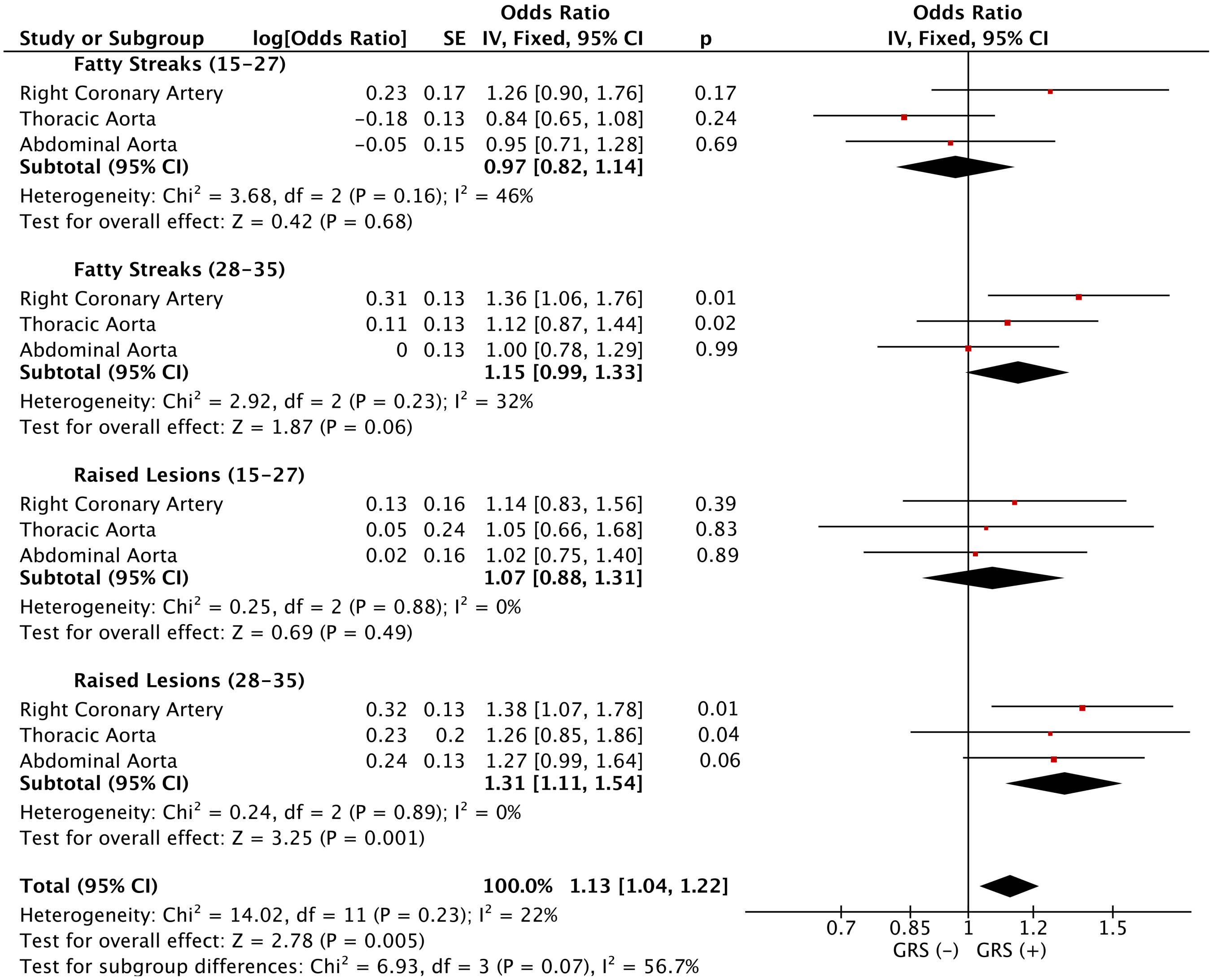

Supplement: S1 File — Table A in S1 File. List of variables used in this study, file names, and embargo dates from the database of genotypes and phenotypes (dbGAP). Table B in S1 File. Association between case-control status of raised lesions or fatty streak for each vascular beds and a weighted GRS composed of 19 SNPs associated with traditional risk factors adjusting for age and sex. Table C in S1 File. Association between case-control status of raised lesions or fatty streak for each vascular beds and a weighted GRS composed of 19 random SNPs from the 57 SNPs associated with clinical CAD and traditional risk factors adjusting for age and sex. Table D in S1 File. Association between case-control status of raised lesions or fatty streak for each vascular beds and a weighted GRS composed of 19 random SNPs from the 38 SNPs associated with clinical CAD only adjusting for age and sex. Table E in S1 File. Association a weighted GRS of 57 SNPs associated with clinical coronary artery disease and case-control status after further filtering by imputation quality r2 (0.3; 0.5; 0.8). Table F in S1 File. Association a weighted GRS restricted to 38 SNPs not associated with traditional risk factors and case-control status after further filtering by imputation quality r2 (0.3; 0.5; 0.8). Table G in S1 File. Association between weighted GRS and case-control status when using genotypes imputed with the Haplotype Reference Consortium. Table H in S1 File. Age and sex adjusted association with case-control status of right coronary raised lesions for each of the 57 single nucleotide polymorphisms used to generate the weighted genetic risk score, ranked by p-value from lowest to highest. Table I in S1 File. Age and sex adjusted association with case-control status of right coronary fatty streak for each of the 57 single nucleotide polymorphisms used to generate the weighted genetic risk score, ranked by p-value from lowest to highest. Table J in S1 File. Age and sex adjusted association with case-control st [file pone.0166994.s001.pdf]
